# Clinical Performance of a Smartphone-Based Sound Amplification Device Versus a Personal Sound Amplification Product in Elders with Mild-to-Moderate Hearing Loss: A Prospective Cohort Study

**DOI:** 10.3390/medicina62030516

**Published:** 2026-03-10

**Authors:** Cheng-Jung Wu, Sheng-Yu Wu, Cheng-Yu Tsai, Arnab Majumdar, Jeffrey Yang, Jinn-Moon Yang, Lok-Yee Joyce Li

**Affiliations:** 1 Department of Otolaryngology, School of Medicine, College of Medicine, Taipei Medical University, Taipei 110, Taiwan; b101090126@tmu.edu.tw; 2 Department of Otolaryngology, Shuang Ho Hospital, Taipei Medical University, Taipei 23561, Taiwan; 3 Ph.D. Degree Program of Biomedical Science and Engineering, National Yang Ming Chiao Tung University, Hsinchu 30010, Taiwan; 4 Department of Industrial Engineering and Industrial Management, National Tsing Hua University, Hsinchu 30010, Taiwan; 5 Department of Civil and Environmental Engineering, Imperial College London, London SW7 2AZ, UK; 6 School of Respiratory Therapy, College of Medicine, Taipei Medical University, Taipei 110, Taiwan; 7 TMU Research Center of Artificial Intelligence in Medicine and Health, Taipei Medical University, Taipei 110, Taiwan; 8 Department of Biology, University of California, Riverside, CA 92521, USA; 9 Institute of Bioinformatics and Systems Biology, National Yang Ming Chiao Tung University, Hsinchu 30010, Taiwan; 10 Department of Biological Science and Technology, National Chiao Tung University, Hsinchu 30010, Taiwan; 11 Department of Environmental and Occupational Medicine, National Taiwan University Hospital Hsin-Chu Branch, Hsinchu County 302058, Taiwan; 12 School of Medicine, College of Medicine, Taipei Medical University, Taipei 110, Taiwan

**Keywords:** sensorineural hearing loss, personal sound amplification product, smartphone, hearing aids, mHealth

## Abstract

*Background and Objectives*: To evaluate the clinical performance of a smartphone-based sound amplification device (SBSAD) compared to a conventional personal sound amplification product (PSAP) in older adults with mild-to-moderate sensorineural hearing loss (SNHL), ad-dressing the need for accessible alternatives given the low adoption of traditional hearing aids. *Materials and Methods*: Forty-nine participants (mean age 68 years) with mild-to-moderate SNHL underwent audiometric testing and subjective evaluation under three conditions: unaided, aided with a commercial PSAP, and aided with an SBSAD (iPhone with wireless earbuds). Primary outcomes included functional gain in sound field thresholds and user ratings of sound quality and acceptability via a custom questionnaire. *Results*: Both devices yielded significant threshold improvements compared to the unaided condition (*p* < 0.001). Mean functional gain was 16.0 dB for the PSAP and 15.3 dB for the SBSAD, with no statistically significant difference (*p* > 0.5). Subjective ratings for sound quality, comfort, cosmetic acceptability, and future willingness to use were comparable between devices (all *p* > 0.05). *Conclusions*: The SBSAD performed equivalently to a traditional PSAP in improving audibility and user satisfaction. Smartphone-based technologies offer a viable, accessible mobile health solution to bridge the gap for older adults who lack conventional hearing aids.

## 1. Introduction

Hearing impairment represents one of the most pervasive and debilitating chronic conditions affecting the geriatric population globally [1]. It is not merely a sensory deficit; rather, it is a multifaceted disorder that significantly restricts communication, impedes social participation, and is independently associated with reduced quality of life, social isolation, depression, and accelerated cognitive decline [1,2,3,4]. The onset of presbycusis, also known as age-related SNHL, is insidious and difficult to detect among the elderly population [b]. Even very slight-to-mild hearing loss can lead to significant difficulties in perceiving sound, especially in challenging listening environments (such as noisy restaurants or family gatherings), which in turn negatively impacts autonomous functions in daily life [1].

In spite of the well-documented prevalence and the associated adverse impacts of hearing impairment, little can be contributed by conservative strategies. Conventional hearing aids (HAs) are the gold-standard treatment, for they substantially improve patients’ hearing abilities and further bring along psychosocial benefits. However, HAs are not widely utilized. Epidemiological surveys showed that less than 20% of adults with measurable hearing loss wear hearing aids. For those with mild hearing impairment, the data is even more worrying, with an estimated adoption rate of less than 1%. The gap between the prevalence of impairment and the rate of treatment acceptance suggests an inescapable flaw in the current model of hearing care services.

The low treatment adoption rate is clinically significant and can be attributed to a list of factors, including high cost, stigmatization, and low accessibility. In fact, as a complicated medical device, conventional hearing aids can only be obtained with physician prescription and professional fitting, followed by a series of follow-up visits for comprehensive assessment and adjustment. Moreover, a pair of hearing aids cost several thousand US dollars on average, which is burdensome to many seniors who rely on a limited pension and lack hearing device insurance coverage.

In response to these market gaps, PSAPs have emerged as a prominent alternative. PSAPs are wearable electronic amplifiers sold over-the-counter directly to consumers.

Unlike hearing aids, they are not regulated as medical devices intended to treat hearing loss, but rather as electronic products for individuals with normal hearing to amplify sounds in specific environments. They typically cost a fraction of the price of prescription HAs. It is estimated that approximately 1.5 million individuals with hearing loss in the United States alone have bypassed traditional clinical routes to use PSAPs or other OTC devices. While early iterations of these devices were simple analog amplifiers, recent technological advances have made some high-end PSAPs functionally comparable to basic digital HAs. For example, a study demonstrated that a high-quality PSAP provided significant improvements in audibility and speech intelligibility, performing nearly on par with an entry-level hearing aid in adults with mild-to-moderate SNHL [5]. Similarly, a large-scale clinical trial reported that a high-end PSAP was non-inferior to both basic and premium HAs for patients with mild-to-moderate hearing loss, suggesting that the gap between medical and consumer audio technology is rapidly closing [6].

The rise of medical PSAP hardware, along with the widespread adoption of smartphones and high-fidelity wireless headphones, has opened a second, and perhaps more revolutionary, path to easily accessible HAs. Modern smartphones possess powerful processing capabilities, allowing for low-latency audio signal processing with performances comparable to dedicated digital signal processing chips in HAs. These devices typically include built-in accessibility features or support downloadable apps that emulate the phone as a hearing aid. A prime example is Apple’s Live Listen and Headphone Accommodations features, which allow wireless headphones (such as AirPods) to amplify ambient sounds based on specific user input. These SBSADs leverage the hardware already owned by the vast majority of the population, potentially and substantially eliminating hardware cost barriers. Furthermore, the form factor of wireless headphones has become widely accepted in public spaces. As they are popular consumer electronics products used for music and calls across all age groups, they are significantly less socially stigmatized than traditional hearing aids [7].

Despite this promising trend toward mobile health hearing solutions, there remains a paucity of formal clinical research evaluating the efficacy of smartphone-based hearing augmentation against established device categories. To our knowledge, no prior published study has directly compared a smartphone-based hearing assistance system—specifically utilizing built-in accessibility algorithms—with a conventional, dedicated PSAP in terms of clinical outcomes and user satisfaction. It remains unclear whether a general-purpose smartphone paired with consumer earbuds can provide an amplification benefit comparable to devices specifically engineered for sound amplification.

Given the potential for smartphone-based solutions to democratize hearing healthcare, we conducted a prospective clinical study to assess how an SBSAD (using standard iPhone and AirPods technology) performs relative to a traditional PSAP in older adults with mild-to-moderate SNHL. We hypothesized that the SBSAD would yield comparable improvements in functional audibility and user satisfaction to the PSAP, thereby validating its potential as a viable entry-level intervention for age-related hearing loss.

## 2. Materials and Methods

### 2.1. Study Design and Participants

We performed a prospective, single institution cohort study at the audiology clinic of Taipei Medical University Shuang Ho Hospital in New Taipei City, Taiwan. The study was conducted in strict adherence to the Declaration of Helsinki. The institutional review board (IRB) of Taipei Medical University approved the study protocol (No. N202012061), and all participants provided written informed consent prior to enrollment.

This study employed a repeated-measures framework, with all patients serving as their own controls. Each patient first underwent a baseline assessment hearing PTA examination without device assistance, followed by post-assessment testing using two different amplification devices. To control for potential order effects, learning effects, or fatigue, the order in which each participant was tested (PSAP or SBSAD first) was randomly assigned using a computer-generated random list. To ensure consistency in device fitting and data collection, all otoscopies were done by a doctor. All device fittings, and outcome measurements were performed by the same senior audiologist.

Based on the World Health Organization (WHO) 2001 World Report on Hearing, the classification defines hearing loss based on the better ear’s pure-tone average (PTA) of 4 frequencies (0.5, 1, 2, and 4 kHz) [8]. The patient’s hearing impairment severity was categorized according to the WHO 2001 World Report on Hearing [8]. Mild hearing impairment was defined as the mean of the hearing thresholds at 500, 1000, 2000, and 4000 Hz > 40 dB hearing loss between 26 and 40 dB HL. Moderate hearing impairment was defined as the mean of the hearing thresholds at 500, 1000, 2000, and 4000 Hz > 40 dB hearing loss between 41 and 60 dB HL [4].

A total of 49 adults were recruited and enrolled in the study. The recruitment targeted the demographic most affected by presbycusis. The inclusion criteria were strictly defined to ensure a homogeneous study population:

1. Age: 21 years or older (targeting the adult and geriatric population).
2. Hearing Status: Diagnosed bilateral, symmetric SNHL.
3. Cognitive Function: A Montreal Cognitive Assessment (MoCA) score of ≥22, ensuring participants had the cognitive capacity to understand instructions, operate the devices, and provide reliable subjective feedback.

Key exclusion criteria included:

1. Any conductive hearing loss component (air-bone gap > 10 dB) or active ear pathology (e.g., otitis media) requiring medical or surgical treatment.
2. Asymmetric hearing loss, defined as a difference of >10 dB in PTA between ears, to avoid confounding factors related to binaural processing deficits.
3. Ear anatomy (including stenosis and atresia) preventing the proper fit of standard earphones.
4. Inability to provide informed consent or complete the questionnaire.

Notably, none of the participants had prior experience with HAs or PSAPs, ensuring that the results reflected the experience of a de novo user group. The cohort consisted of 20 males (40.8%) and 29 females (59.2%), with a mean age of 68.0 years (SD 9.4; range 55–80 years).

### 2.2. Devices and Interventions

Participants were evaluated under two aided conditions:

1. Personal Sound Amplification Product: This study used a standardized, commercially available behind-the-ear style personal sound amplifier. This device represents a typical high-quality PSAP on the market. It features basic digital signal processing capabilities, including noise reduction and feedback cancelation. Audiologists adjusted the volume and gain settings to provide appropriate amplification based on the participants’ audiograms.
2. Smartphone-Based Sound Amplification Device: This system consisted of an Apple iPhone (running iOS 14) paired with Apple AirPods (2nd generation).

We utilized the Headphone Accommodations accessibility feature, which is FDA-cleared for providing hearing assistance. The setup process involved the audiologist inputting the participant’s specific audiometric thresholds into the Apple Health app. The algorithm then automatically generated a personalized amplification profile (custom transparency mode) compensating for the specific frequency deficits. During testing, the “Live Listen” function was activated, using the iPhone’s microphone to capture ambient sound, process it according to the profile, and stream it to the AirPods. The iPhone was positioned approximately 30 cm in front of the participant to simulate a typical conversation or listening scenario.

### 2.3. Outcomes and Statistical Analysis

Outcomes were categorized into objective audiometric data and subjective user-reported measures.

1. Audiometric Testing: Baseline pure-tone audiometry was conducted in a sound-treated booth meeting ANSI standards. Air-conduction thresholds were obtained at 0.5, 1, 2, 4, and 8 kHz for each ear to establish the degree of hearing loss.
2. Aided Sound-Field Testing (Functional Gain): The primary objective outcome measure was the functional gain measured in the sound field. Participants sat 1 m from the calibrated speaker at 0 degrees azimuth. Warble tones were used to reduce standing wave interference. Thresholds were measured at 0.5, 1, 2, and 4 kHz under three conditions: no device, with PSAP, and with SBSAD. Functional gain was calculated as the decibel difference between the unequipped and device-equipped thresholds at each frequency. The average values for the four frequencies (0.5, 1, 2, and 4 kHz) were then derived for analysis.
3. Subjective Questionnaire: Following the sound field test, participants completed a custom-designed structured questionnaire to assess their subjective experience. Scoring was done using a 10-point Likert scale (1 = very poor, 10 = excellent), covering five domains.

- Sound Quality: Naturalness and clarity of the amplified sound.
- Wearing Comfort: Physical sensation of the device in/on the ear.
- Aesthetics/Appearance: Cosmetic acceptability and willingness to wear in public.
- Ease of Use/Portability: Convenience of operation and transport.
- Overall Acceptance: Likelihood of future adoption. Participants also indicated a forced-choice preference: “Prefer PSAP,” “Prefer SBSAD,” or “No Preference.”

#### Statistical Analysis

Data were analyzed using commercially available statistical software. Descriptive statistics (mean ± standard deviation) were generated for demographic and outcome variables. The normality of data distribution was assessed using the Shapiro–Wilk test. Paired *t*-tests were utilized to compare continuous variables (sound-field thresholds) between the two devices and against the unaided baseline. For ordinal data derived from the Likert scale questionnaires, the Wilcoxon signed-rank test was employed to verify the robustness of the findings. A *p*-value of <0.05 was considered statistically significant.

## 3. Results

### 3.1. Participant Characteristics

The study successfully completed the protocol for all 49 enrolled participants (Table 1). The demographic profile was representative of the general population seeking assistance for age-related hearing loss. The hearing loss severity was categorized as mild in 12 participants (24%) and moderate in 37 participants (76%). The mean unaided baseline PTA was 48.1 dB HL. All participants demonstrated normal cognitive function (MoCA ≥ 22), validating the reliability of the subjective feedback collected.

A total of 78 students were obligated to attend this lesson. However, three of them were late for class; four of them left the classroom before the lesson ended; and there were five students who did not sign the consent form. In short, there were 66 students included in this study. Of the above mentioned 66 dental students, 36 wore a mask and 30 did not. All students brought their smartphones to class and they were required to put them on the desk during class.

Among the 66 dental students, they were observed to have touched their faces 624 in 1 h, with an average of 9.45 ± 8.41 touches per hour Table 1. Of the 66 students, they were also observed to have touched their smartphones 454 in an hour, with an average of 6.88 ± 6.25 touches per hour Table 1.

### 3.2. Hearing Threshold Improvement (Audiometric Outcomes)

Both amplification modalities demonstrated robust clinical efficacy in improving audibility. As detailed in Table 2, the mean unaided sound-field threshold for the cohort was 48.1 ± 8.5 dB HL. Upon application of the devices, thresholds improved significantly.

- The PSAP reduced the mean threshold to 32.1 ± 7.4 dB HL, yielding a mean functional gain of 16.0 dB (*p* < 0.001 vs. unaided).
- The SBSAD reduced the mean threshold to 32.8 ± 7.1 dB HL, yielding a mean functional gain of 15.3 dB (*p* < 0.001 vs. unaided).

When comparing the two aided conditions directly, there was no statistically significant difference in the resultant hearing thresholds (Difference = 0.7 dB; *p* > 0.5). This indicates that the smartphone-based system provided amplification power equivalent to the dedicated PSAP device for determining soft sounds in a quiet environment.

### 3.3. Questionnaire: User Satisfaction and Preference

- Subjective data mirrored the objective findings, revealing high satisfaction rates for both modalities with no significant superiority for either device (Table 3). In terms of overall preference, the cohort was divided. Approximately 35% (*n* = 17) slightly preferred the SBSAD, citing its modern appearance and integration with a device they already owned. Conversely, 31% (*n* = 15) preferred the PSAP, often highlighting its “always-on” simplicity and lack of need for pairing or app management. The remaining 35% (*n* = 17) expressed no strong preference. Qualitative feedback suggested a trade-off: the SBSAD was less stigmatizing, whereas the PSAP was perceived as simpler for those less comfortable with smartphone interfaces.

## 4. Discussion

In recent years, there has been a rapid evolution in the development of mobile health services. Smartphone-based audiometry is considered as a fast, easy, and reliable technique for cost-effective screening of hearing impairments. Free apps can be downloaded from the Android market and installed in any smartphone. In our previous study, the sensitivity and the specificity of the smartphone-based air conduction audiometry test were 0.92and 0.76 [9]. The sensitivity and the specificity of the smartphone-based bone conduction audiometry test were 0.64 and 0.71 [10]. Hearing impairment is a frequent human sensory impairment. It was estimated that over fifty percent of elders experience hearing impairment in the United States. Several hearing impairment-related factors are detectable through screening; thus, further deterioration can be avoided. Once hearing impairment is identified, it is essential that it is addressed as early as possible and in an appropriate manner to mitigate any adverse impact [11]. Early identification and treatment of hearing impairment is the key to effective management [12].

Data from Margaret I. Wallhagen’s study document the pervasive nature of stigma in relationship to hearing loss and HAs and the close association of this stigma with ageism [12]. There are many strategies to minimize stigma, including valuing hearing loss by enhancing its assessment and treatment as well as emphasizing the importance of remaining actively engaged, which are needed to help older adults and their families maintain positive physical and cognitive functioning [12]. iPhone with AirPods is actively destigmatizing hearing loss by transforming assistive technology into stylish, mainstream consumer tech. Their adoption allows users to navigate noisy environments discreetly without the stigma associated with traditional HAs. This shift towards invisible disability support boosts confidence, as users feel fashionable rather than exposed [13].

Given the economic barriers to hearing care, the performance equivalence of SBSADs and PSAPs has significant clinical implications. For most of the global population, traditional HAs remain prohibitively expensive. Our findings confirm that mobile health is an immediate and readily available intervention. By utilizing hardware that many users already own, SBSADs effectively decouple the cost of the amplifier from the cost of the hearing solution. This is consistent with the findings of Lin et al., who pointed out that smartphone earphones can be used as effective PSAPs [14]. Furthermore, with recent regulatory changes (such as the establishment of the OTC category for nonprescription HAs in the United States), the line between medical devices and consumer audio devices is becoming blurred. Our findings support the safety and effectiveness of this change, showing that properly configured consumer devices do more than just amplify noise, they can provide target gain comparable to basic HAs.

A key obstacle to hearing aid adoption is the stigma of senescence and the association of HAs with aging and disability. Manchaiah et al. emphasized that devices that are directly accessible to consumers and resemble medical HAs often face the same stigma problem [7]. However, this study used wireless headphones, which have gained widespread social acceptance. In our results, SBSADs scored higher than PSAPs (7.4 vs. 7.1) on the appearance item, although the difference was not statistically significant. Qualitative feedback showed that the reason why subjects felt less comfortable wearing headphones was that everyone has headphones. This suggests that the appearance of mobile health devices may help bypass psychological barriers that prevent the elderly from seeking help and may potentially act as a gateway device, encouraging early intervention in the progression of hearing loss. Our results echo the work of Choi et al. and Cho et al., who established that high-end PSAPs can rival HAs [5,6].

We further demonstrated that non-dedicated hardware (such as mobile phones) can also compete with dedicated PSAPs. This may be attributed to the powerful processing capabilities of modern smartphones, which allow for complex algorithms (such as compression, noise gate, and frequency trimming) with extremely low latency. Unlike previous hearing aid apps that were plagued by audio latency, the integration of features such as Live Listen at the operating system level ensures a seamless auditory experience. It has been proven that the preset adaptation results based on audiograms on mobile phones can keep pace with devices specifically designed for PSAPs, which demonstrates the maturity of consumer-grade audio processing technology [15].

The divergence in user preferences (35% preferred SBSAD vs. 31% preferred PSAP) highlights that there is no one-size-fits-all solution for hearing needs. While SBSAD has advantages in aesthetics and cost, it also adds complexity; users must manage Bluetooth connectivity, the battery life of both devices (phone and headphones), and the user interface of the application. In contrast, PSAP represents a set-and-forget ease of use, which is preferred by some older adults. This finding is consistent with the study who emphasized that providing a variety of technology options is crucial to meeting patient needs [16]. SBSAD may be ideal for tech-literate active older adults, while PSAP remains a strong option for users who prioritize ease of use.

Several limitations of this study should be considered. First, the sample size (N = 49) is relatively small, although sufficient for preliminary comparisons. Second, the tests were conducted in a soundproof room with acoustic processing. While this provides clean data on hearing ability, it does not reflect real-world challenges. As suggested by the other study, future research must include speech-in-noise testing to determine whether smartphone microphones can handle background noise as effectively as directional microphones in dedicated HAs [17]. Third, we used a specific ecosystem (Apple); the results may not be generalizable to the fragmented Android market due to the significant differences in audio latency between different Android devices [18].

The most common complaint that people have about their hearing loss is difficulty understanding speech, especially when listening in a noisy environment [19]. While this study provides promising evidence for the clinical equivalence of SBSADs and PSAPs, several limitations must be acknowledged. First, our assessment relied primarily on tonal functional gain measured in a controlled, quiet sound-field environment. As noted by previous researchers, pure-tone thresholds do not fully capture vocal gain or the complexities of speech perception in real-world settings. We did not measure vocal gain or speech perception metrics, which are critical for evaluating real-world communication efficacy. We plan to incorporate comprehensive speech-in-noise testing in future research

At last, this study did not specifically account for advanced auditory phenomena such as recruitment or hyperacusis, which are common in patients with SNHL and often complicate the fitting of amplification devices. Unlike medical-grade HAs that offer sophisticated wide dynamic range compression and precise maximum power output limitations, current consumer-grade SBSADs may have more limited capacity to manage these recruitment-related comfort issues. This study did not include a control group using prescription medical-grade HAs; thus, while we can claim equivalence to PSAPs, we cannot claim equivalence to premium medical HAs.

## 5. Conclusions

In this cohort of older adults with mild-to-moderate SNHL, an SBSAD utilizing wireless earbuds performed equivalently to a traditional personal sound PSAP in improving hearing thresholds and user satisfaction. Both devices provided significant functional gain in a quiet environment. These findings suggest that SBSADs represent a promising, accessible, and socially acceptable mHealth solution for the large population of individuals who remain untreated due to the cost or stigma of conventional HAs. As consumer technology continues to intersect with healthcare, the wider adoption of such smartphone-based technologies could play a pivotal role in bridging the gap in hearing care for the elderly.

## Figures and Tables

**Table 1:** Participant demographics and baseline hearing characteristics (*n* = 49).

Characteristic	Value
Age, mean (years)	68.0 (SD 9.4)
Sex distribution	20 male (40.8%), 29 female (59.2%)
Hearing loss severity	12 mild (24%); 37 moderate (76%)
Baseline PTA (0.5–4 kHz), mean	48 dB HL (mild SNHL subgroup 36; moderate SNHL subgroup 52)
Cognitive status	MOCA score ≥22 for all (normal cognition)

Abbreviations: PTA = pure-tone average; dB HL = decibels hearing level; MoCA = Montreal Cognitive Assessment.

**Table 2:** Unaided and Aided Sound-Field Thresholds (Functional Gain) for PSAP vs. SBSAD (average across 0.5–4 kHz tones, in dB HL).

Hearing Condition	Mean Threshold (SD)	Functional Gain vs. Unaided	*p*-Value (Unaided vs. Aided)
Unaided (no device)	48.1 dB HL (±8.5)		
Aided with PSAP	32.1 dB HL (±7.4)	16.0 dB (gain)	*p* < 0.001
Aided with SBSAD	32.8 dB HL (±7.1)	15.3 dB (gain)	*p* < 0.001

**Table 3:** Mean Questionnaire Ratings for PSAP vs. SBSAD (scale: 1 = very poor, 10 = excellent).

Aspect Rated	PSAP Mean (SD)	SBSAD Mean (SD)	*p*-Value (PSAP vs. SBSAD)
Overall sound quality	7.0 (±1.5)	6.4 (±1.8)	0.19
Wearing comfort	7.3 (±1.6)	7.0 (±1.7)	0.35
Appearance (willingness to wear)	7.1 (±1.9)	7.4 (±1.6)	0.27
Portability & ease of use	7.6 (±1.3)	7.4 (±1.4)	0.45
Overall acceptance for future use	7.1 (±1.8)	7.1 (±1.6)	0.99

## Data Availability

The data that support the findings will be available on request under the corresponding author’s e-mail: b101102173@tmu.edu.tw.
